# Supplementary figures and images for: Zebra stripes, tabanid biting flies and the aperture effect
Source: Proc Biol Sci. 2020 Aug 19;287(1933):20201521. doi: 10.1098/rspb.2020.1521 (PMC7482270; doi:10.1098/rspb.2020.1521)

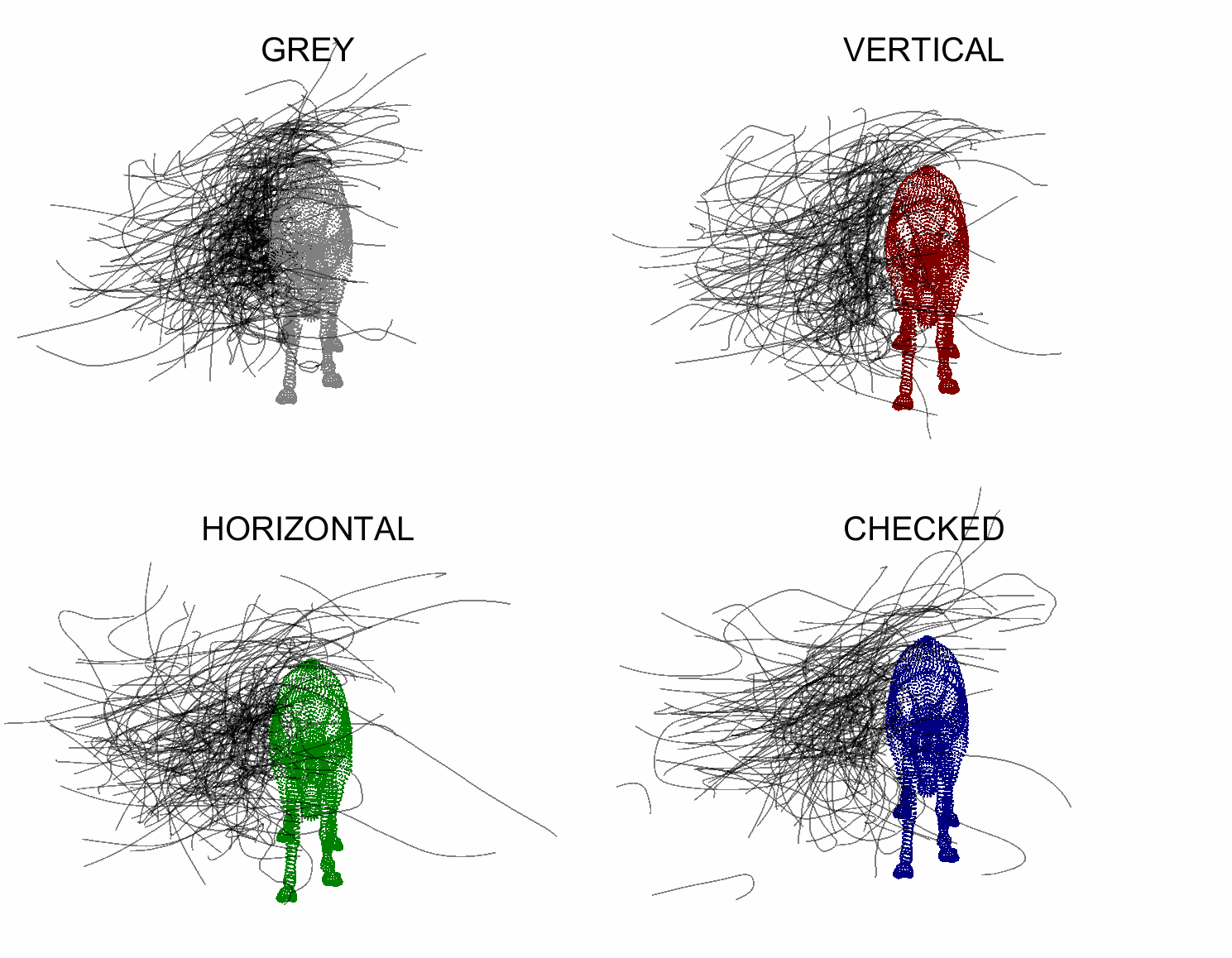

Supplement: Supplementary movie S3. [file rspb20201521supp1.gif]
