# Supplementary material for: Zebra stripes, tabanid biting flies and the aperture effect
Source: Proc Biol Sci. 2020 Aug 19;287(1933):20201521. doi: 10.1098/rspb.2020.1521 (PMC7482270; doi:10.1098/rspb.2020.1521)
Supplement: Supplementary information for: Zebra stripes, tabanid biting flies, and the aperture effect [file rspb20201521supp2.pdf]

Supplementary information for: Zebra stripes, tabanid biting flies, and the aperture effect

Martin J. How[1*], Dunia Gonzales[1], Alison Irwin[1], and Tim Caro[1,2]

1. School of Biological Sciences, University of Bristol, 24 Tyndall Avenue, Bristol BS8 1TQ, UK
2. Center for Population Biology, University of California, Davis, CA 95616, USA

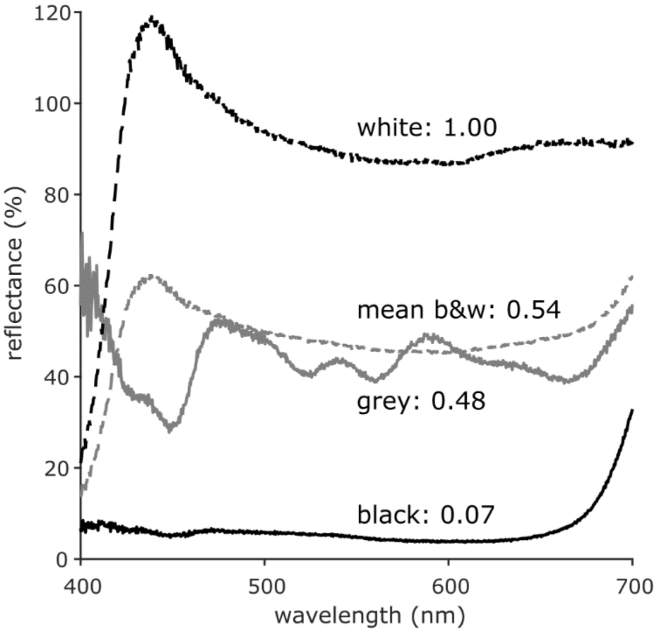

Supplementary figure S1. Spectral reflectance of pattern components on the printed fabric. Reflectance values are calculated relative to Spectralon white standard (OceanOptics, Largo, USA). Overall intensity relative to the white fabric is noted near each line. Dashed grey line is the mean reflectance of black and white components combined. Note that the >100% peak for white fabric is likely due to fluorescence in this part of the spectrum.

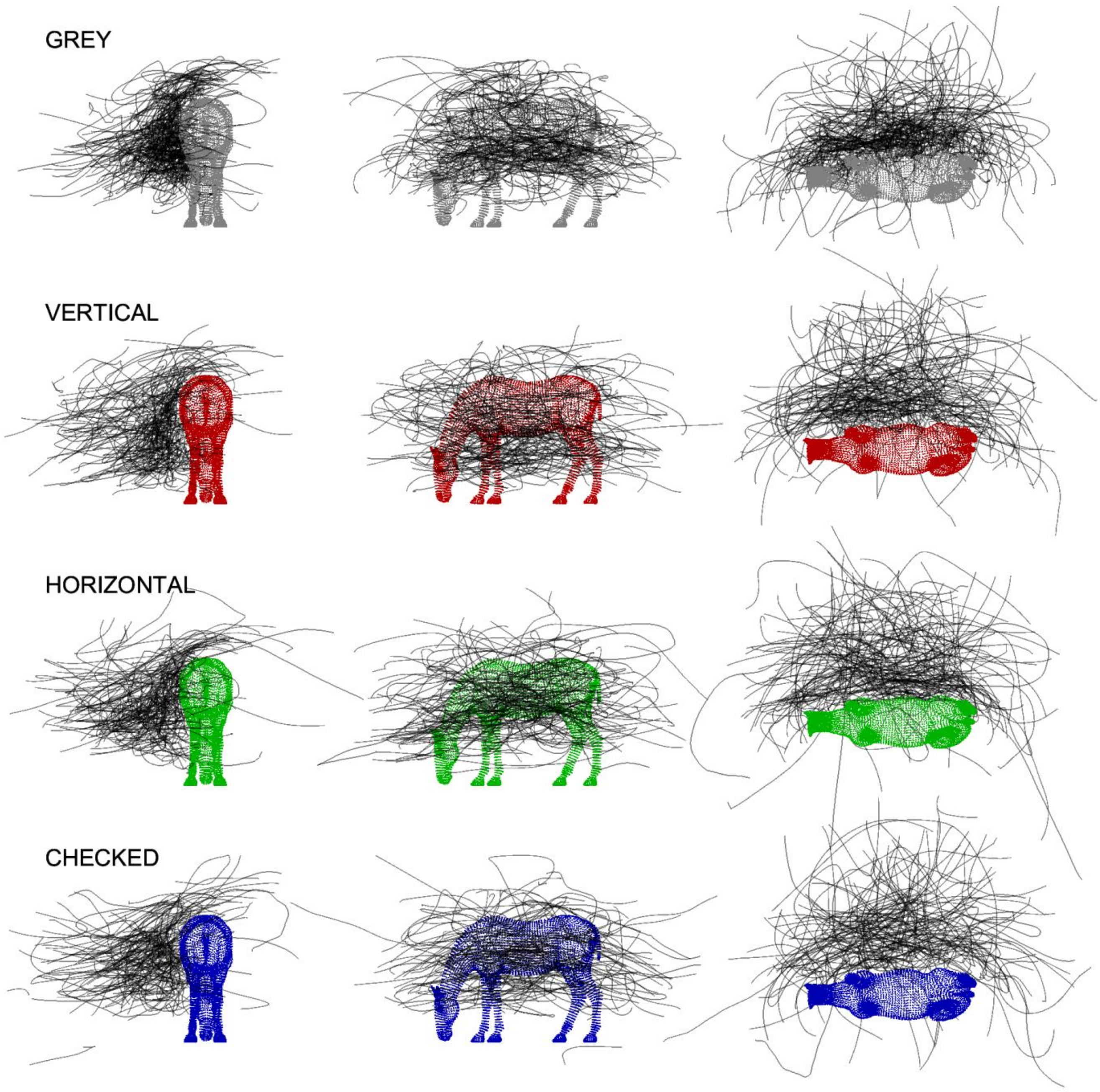

Supplementary figure S2. Horsefly flight trajectories for all four rug types viewed from three different angles: left = frontal; middle = lateral; right = dorsal. N = 101-107 fly trajectories per rug type.

Supplementary movie S3. Animated version of the data in Fig S2.

Supplementary table S1. Statistical differences between fly approach speeds to the four different rug types at each distance interval (Kruskal-Wallis with multicompare post hoc comparison with Bonferroni correction for multiple tests between rugs) at each distance bin. Results smaller than p=0.05 are highlighted in green.

**Distance (m)**		**0.05**	**0.15**	**0.25**	**0.35**	**0.45**	**0.55**	**0.65**	**0.75**	**0.85**	**0.95**	**1.05**
vs.												
grey	vertical	1.000	0.014	0.028	<0.001	<0.001	0.018	0.617	0.772	1.000	1.000	1.000
grey	horizontal	0.157	<0.001	<0.001	<0.001	<0.001	<0.001	0.003	0.006	0.165	0.049	1.000
grey	checked	1.000	0.010	<0.001	<0.001	0.003	<0.001	0.149	0.348	1.000	0.886	1.000
vertical	horizontal	1.000	0.077	0.018	1.000	1.000	0.110	0.335	0.322	0.084	0.016	0.080
vertical	checked	1.000	1.000	1.000	1.000	1.000	1.000	1.000	1.000	1.000	0.591	0.524
horizontal	checked	0.508	0.226	0.556	1.000	0.624	1.000	1.000	0.932	1.000	1.000	1.000
